# 2533. Cerebrospinal Fluid Penetration of Cefazolin and Flucloxacillin in Children

**DOI:** 10.1093/ofid/ofad500.2151

**Published:** 2023-11-27

**Authors:** Alison Boast, Alice Lei, Kiera Harwood, Gena Gonis, Amanda Di Carlo, Andrew J Daley, Jacobus Ungerer, Brett McWhinney, Nigel Curtis, Amanda Gwee

**Affiliations:** Royal Children's Hospital Melbourne, Melbourne, Victoria, Australia; Murdoch Children's Research Institute, Melbourne, Victoria, Australia; Murdoch Children's Research Institute, Melbourne, Victoria, Australia; Royal Children's Hospital Melbourne, Melbourne, Victoria, Australia; Royal Children's Hospital, Melbourne, Victoria, Australia; Royal Children's Hospital Melbourne, Melbourne, Victoria, Australia; Pathology Queensland, Melbourne, Victoria, Australia; Pathology Queensland, Melbourne, Victoria, Australia; The Royal Children's Hospital, Melbourne, Victoria, Parkville, Victoria, Australia

## Abstract

**Background:**

Effective treatment of methicillin-susceptible *Staphylococcus aureus* meningitis requires antimicrobial penetration into the CSF. Anti-staphylococcal penicillins, including flucloxacillin, are generally recommended as first-line treatment. However, some evidence suggests that the first-generation cephalosporin, cefazolin, may be an appropriate alternative. We investigated the CSF concentration of cefazolin and flucloxacillin in children.

**Methods:**

CSF samples from children aged 0-18 years treated with cefazolin or flucloxacillin were collected when there was sample remaining after diagnostic tests were completed. Unbound antibiotic concentrations of cefazolin and flucloxacillin were measured.

**Results:**

Overall, 222 CSF samples were collected from 163 children, median age 12 months (range 0.0-17.5 years). Of these, 157 were collected while the antibiotic was at steady state. For cefazolin, children ≤1 year had a higher median CSF concentration compared with those >1 year (1.8 versus 0.5 mg/L, p< 0.001), whereas for flucloxacillin the concentration was similar between the two age groups (0.2 versus 0.1 mg/L, p=0.9). In infants aged ≥ 1 month to 1 year, the CSF concentration of both antibiotics was higher in those with pleocytosis (WCC >5 x10^6^/L) than those without (flucloxacillin 0.2 versus 0.065 mg/L, cefazolin 5.6 versus 1.06 mg/L). Notably, only those aged ≤1 year receiving cefazolin (7/12; 58%) achieved steady state CSF concentrations above the CLSI breakpoint for sensitive *S. aureus* (MIC ≤2 mg/L).

**Conclusion:**

CSF penetration of cefazolin was higher in younger infants and for both antibiotics was higher in those with pleocytosis. In most children, the CSF concentrations in the CSF for either cefazolin or flucloxacillin were not above the *S. aureus* MIC.

**Disclosures:**

**All Authors**: No reported disclosures

